# Genetic Detection of Lint Percentage Applying Single-Locus and Multi-Locus Genome-Wide Association Studies in Chinese Early-Maturity Upland Cotton

**DOI:** 10.3389/fpls.2019.00964

**Published:** 2019-08-02

**Authors:** Junji Su, Caixiang Wang, Fushun Hao, Qi Ma, Ji Wang, Jilian Li, Xinzhu Ning

**Affiliations:** ^1^Gansu Provincial Key Laboratory of Aridland Crop Science, College of Life Science and Technology, Gansu Agricultural University, Lanzhou, China; ^2^State Key Laboratory of Cotton Biology, Institute of Cotton Research of Chinese Academy of Agricultural Sciences, Anyang, China; ^3^State Key Laboratory of Cotton Biology, Henan Key Laboratory of Plant Stress Biology, College of Life Science, Henan University, Kaifeng, China; ^4^Cotton Research Institute, Xinjiang Academy of Agricultural and Reclamation Science, Shihezi, China

**Keywords:** upland cotton, lint percentage, early maturity, genome-wide association studies, quantitative trait nucleotides, candidate genes

## Abstract

Upland cotton (*Gossypium hirsutum* L.) is the most important source of natural fiber in the world. Early-maturity upland cotton varieties are commonly planted in China. Nevertheless, lint yield of early-maturity upland cotton varieties is strikingly lower than that of middle- and late-maturity ones. How to effectively improve lint yield of early maturing cotton, becomes a focus of cotton research. Here, based on 72,792 high-quality single nucleotide polymorphisms of 160 early-maturing upland cotton accessions, we performed genome-wide association studies (GWASs) for lint percentage (LP), one of the most lint-yield component traits, applying one single-locus method and six multi-locus methods. A total of 4 and 45 significant quantitative trait nucleotides (QTNs) were respectively identified to be associated with LP. Interestingly, in two of four planting environments, two of these QTNs (A02_74713290 and A02_75551547) were simultaneously detected *via* both one single-locus and three or more multi-locus GWAS methods. Among the 42 genes within a genomic region (A02: 74.31–75.95 Mbp) containing the above two peak QTNs, *Gh_A02G1269, Gh_A02G1280*, and *Gh_A02G1295* had the highest expression levels in ovules during seed development from 20 to 25 days post anthesis, whereas *Gh_A02G1278* was preferentially expressed in the fibers rather than other organs. These results imply that the four potential candidate genes might be closely related to cotton LP by regulating the proportion of seed weight and fiber yield. The QTNs and potential candidate genes for LP, identified in this study, provide valuable resource for cultivating novel cotton varieties with earliness and high lint yield in the future.

## Introduction

As one of the most important cash crops, upland cotton (*Gossypium hirsutum* L.) is widely cultivated around the world and supplies the most raw materials for the textile industry. China is one of the largest nations producing cotton fiber with the highest per unit area yield in the world (Dai and Dong, 2014). At present, the increased average yield of cotton is mainly ascribed to the application of many improved varieties as well as precise farming technologies in China (Dai and Dong, 2014; Feng et al., 2017). It is well-known that upland cotton can be classified into early-, middle-, and late- maturity varieties based on the duration of their growth periods. Early-maturity upland cotton features early flowering, early boll-opening, and short and compact plant architecture (Gwathmey et al., 2016). It is appropriate for the precise cultivation or production patterns including double cropping, high-density planting, and mechanical harvesting (Yu et al., 2005; Song et al., 2015; Feng et al., 2017; Su et al., 2018). Therefore, the early-maturity cotton varieties are becoming increasingly important in Chinese cotton production. However, cotton fiber yield of early-maturity varieties was strikingly lower than that of middle- and late- maturity ones. Hence, improving fiber yield is a major goal in Chinese early-maturity cotton breeding practice.

Over the past 40 years, traditional breeding strategy has played important roles in early-maturity cotton breeding in China, and a series of early-maturity cotton varieties like “Liaomian,” “Xinluzao,” and “Zhongmiansuo” had been bred by using hybridization and backcrossing. However, due to the striking negative correlation between high yield and early maturity, it is hard to cultivate varieties with high lint yield and earliness by means of traditional breeding strategy (Song et al., 2005; Fan et al., 2006; Su et al., 2016b). LP is one of the most lint-yield component traits in cotton, and its heritability and stability are high, even though it is influenced by different natural environmental conditions (Su et al., 2016a). Therefore, illuminating the molecular mechanisms underlying LP is very essential for improving lint yield in early-maturity cotton breeding.

Lint percentage was mainly controlled by many QTL, which can be detected by linkage analysis and association mapping. Over the past two decades, a number of QTL for LP had been identified *via* linkage analysis in upland cotton (Zhang et al., 2005; Abdurakhmonov et al., 2007; Shen et al., 2007; Liu et al., 2012; Yu et al., 2013; Wang et al., 2014). Compared with QTL mapping based on linkage analysis for LP, detections of marker-trait association with LP in upland cotton were relatively few. For example, a few QTNs associated with LP had been identified *via* GWASs in upland cotton (Su et al., 2016a; Fang et al., 2017; Huang et al., 2017; Ma et al., 2018). Among these association studies, single-locus GWAS (SL-GWAS) models, based on the MLM, were utilized. Nevertheless, multi-locus GWAS (ML-GWAS) models were seldom applied to dissect genetic basis for cotton LP.

In general, multiple tests and Bonferroni correction are made to reduce the false positive rate in the SL-GWAS experiments. These may result in exclusion of some important locus-trait associations because of the strict significance-test criterion. As an excellent complement to SL-GWAS, the new ML-GWAS models, which are beneficial to identifying more loci associated with the target traits, were applied in the past 10 years, because the stringent Bonferroni correction was not needed using these models. In the last 3 years, researchers had developed six new multi-locus GWAS (ML-GWAS) models including mrMLM (Wang et al., 2016), ISIS EM-BLASSO (Tamba et al., 2017), FASTmrEMMA (Wen et al., 2018), pLARmEB (Zhang et al., 2017), FASTmrMLM (Tamba and Zhang, 2018), and pKWmEB (Ren et al., 2018). Some of them had also been used in upland cotton recently (Hou et al., 2018; Su et al., 2018). Moreover, application of a combination of both SL- and ML-GWAS methods was reported to be useful for improving accuracy of GWAS results (Li et al., 2018; Xu et al., 2018).

In this study, to decrypt genetic basis for LP in Chinese early-maturity upland cotton, we integrated one single-locus method (MLM) and six multi-locus methods (mrMLM, ISIS EM-BLASSO, FASTmrEMMA, pLARmEB, FASTmrMLM, and pKWmEB), and performed GWAS analyses for LP using 72,792 high-quality SNPs of 160 early-maturing cotton accessions. The favorable allelic variations of the peak QTNs and the potential candidate genes for objective trait were further identified. This effort will lay the basis for breeding excellent upland cotton varieties with high lint yield and earliness in the future.

## Materials and Methods

### Plant Materials

An association panel which comprises 160 Chinese early-maturity upland cotton accessions (Supplementary Table S1) was reported in our previous study (Su et al., 2018). These accessions were collected from the germplasm gene bank of the Institute of Cotton Research of Chinese Academy of Agricultural Sciences (ICR-CAAS). Based on cotton growing areas in China, the germplasms were divided into three categories: the Yellow River Region (YRR, 81 accessions), the NIR (58 accessions) and the NSEMR (21 accessions), respectively. All the early-maturity cotton accessions, which were planted for more than 3 years, had relatively wide LP distributions ranging from 28 to 48%.

### Phenotyping and Data Analysis

A total of 160 early-maturity upland cotton accessions were examined under four planting conditions: two locations: Anyang (AY), Henan, China (36.13° N, 114.80° E) and Shihezi (SHZ), Xinjiang, China (44.52° N, 86.02° E); 2 years: 2014 and 2015. Four planting environments were respectively designated as AY-2014, AY-2015, SHZ-2014, and SHZ-2015. All the trials adopted a randomized complete block design and had three replications. Each accession was planted in a plot with 4.00 m^2^ (0.80 m × 5.00 m) in AY-2014 and AY-2015, whereas each accession was sown in a plot with about 3.00 m^2^ (0.76 m × 4.00 m) in SHZ-2014 and SHZ-2015. Both experiments, at SHZ, were conducted with conditions of drip irrigation under plastic film, while two trials at AY were furrow irrigated as required. The field management measures were conducted according to local agronomic practices.

After maturing, 20 spontaneously opened bolls were gathered from middle part of the plants from each accession in each replicate for 2 years. For each cotton sample, 20-BW and their LW were measured by electronic balance. LP was calculated according to the formula of LP (%) = LW (g)/BW (g) × 100%. The ANOVA for LP phenotypic data was conducted using SPSS 24.0 software.

### Marker-Trait GWAS

The approaches of DNA extraction, SLAF-seq, and SNP calling were reported in detail in the previous study (Su et al., 2018). In the light of filtering criterion of missing rate < 20% and MAF ≥ 0.05, a total of 72,792 high-quality SNPs were obtained and used to the following analysis (Su et al., 2018). In this study, both SL-GWAS and ML-GWAS were respectively conducted for four individual environments. The SL-GWAS was performed in MLM (PCs + K) by using the Tassel 5.2 program (Bradbury et al., 2007). Its *P*-value threshold for significant association was 6.87E-05 (5/total SNPs used), that is to say, those with -lg*P* ≥ 4.16 were considered the significant marker-trait associations. The six ML-GWAS methods including the mrMLM (Wang et al., 2016), ISIS EM-BLASSO (Tamba et al., 2017), FASTmrEMMA (Wen et al., 2018), pLARmEB (Zhang et al., 2017), FASTmrMLM (Tamba and Zhang, 2018), and pKWmEB (Ren et al., 2018) were used in this study. For the above six ML-GWAS methods, all the parameters were set to defaults, and the PCs and K covariates were added to the model. All the significant-association thresholds were set to LOD = 3.00. The SNPs loci, which satisfied the above criterion, were regarded as the QTNs with the significant marker-trait associations.

### Analysis of Favorable Allelic Variations

For the peak QTNs, the phenotypic value of each allelic variation was estimated by the phenotypic values for the accessions with each type of QTN. The favorable allelic variations of the QTNs were subsequently identified according to the breeding objectives of target trait. Box plots for the relative phenotypic values were performed using R software.

### Prediction of Potential Candidate Genes

The physical positions of the pivotal locus-trait associations were applied to identify putative candidate genes in the *Gossypium hirsutum* L reference genomes v1.1 (Zhang et al., 2015). According to LD decay distance and the positions of the significant QTNs, we determined the prediction intervals, which contained the potential candidate genes. Then, the genes, which were distributed in these regions, were picked out, and their expression levels were estimated by a RNA-seq. The RNA-seq datasets of 17 cotton tissues [root, stem, leaf, ovules from -3, -1, 0, 1, 3, 5, 10, 20, 25, and 35 DPA, and fibers from 5, 10, 20, and 25 DPA of *G. hirsutum* “TM-1”] were available on the NCBI SRA database^1^ (Zhang et al., 2015). Normalized FPKM values were reckoned to show the gene expression levels. The mean of the two biological replicates was considered as the final FPKM values. Heatmaps of the putative candidate gene expression styles were drawn using the R package “pheatmap.” The biological functions of putative candidate genes were annotated by GO items on the cotton website^2^.

## Results

### Phenotypic Variation in All Accessions

The LP phenotypic values among these 160 upland cotton accessions were used for the variation analysis across four environments. In the four experiments, the mean LP values (± SD) were 40.96 ± 2.77, 39.78 ± 3.19, 41.77 ± 2.60, and 42.05 ± 2.46% in AY-2014, AY-2015, SHZ-2014, and SHZ-2015, respectively. In AY-2015, the LP values ranged from 28.58 to 47.22%, with the maximum coefficient of variation (CV) of 8.01%; whereas in SHZ-2015, the LP had the minimum variation ranging from 31.59 to 47.03%, with a smallest CV value of 5.84% (Table 1). The phenotypic evaluation results indicate that the early-maturity upland cotton varieties have broad variation of LP among the 160 accessions.

**Table 1:** Phenotypic distribution range of lint percentage (LP) of 160 early-maturity upland cotton accessions.

Environments	AY-2014	AY-2015	SHZ-2014	SHZ-2015
Mean (%)	40.96	39.78	41.77	42.05
Max (%)	45.75	47.22	46.09	47.03
Min (%)	30.31	28.58	30.60	31.59
*SD* (%)	2.77	3.19	2.60	2.46
CV (%)	6.77	8.01	6.22	5.84

*Max, maximum; Min, minimum; SD, standard deviation; CV, coefficient of variation; AY, Anyang; SHZ, Shihezi.*

**FIGURE 1:**
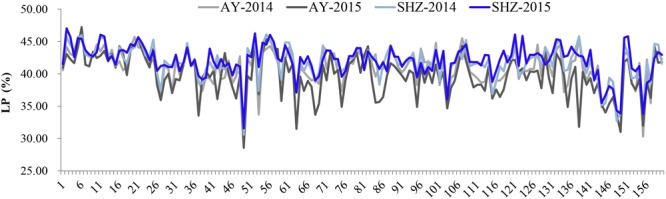
Phenotypic distributions of lint percentage (LP) of 160 early-maturity upland cotton accessions in four growing environments.

To examine whether LP variances were significantly influenced by the external environments, comparative analyses were conducted among the LP values across the four different environments. We observed that the LP values at SHZ were obviously higher than those at AY among these 160 accessions (Figure 1). Furthermore, the ANOVA showed that there were significant differences (*P* < 0.001) for LP among genotypes (G), environments (E), and the G × E interactions (Supplementary Table S2). These results imply that the LP is clearly affected by the external environmental conditions.

### Marker-Trait Associations Based on Both SL-GWAS and ML-GWAS

In our previous study, on the basis of 72,792 SNPs, the PCs and K values of the association panel were estimated, and the population structure of these 160 upland cotton accessions was separated into three subpopulations by two means of principal component analysis (PCA) and the phylogenetic tree (Su et al., 2018). In the study, in order to detect accurately the genetic variations associated with LP trait in Chinese early-maturity upland cotton, we performed simultaneously SL-GWAS and ML-GWAS.

Applying the MLM of SL-GWAS, four significant QTNs for LP were detected, and two, one, and one of them were located on chromosomes A02, A07, and A12, respectively (Table 2). Among these four QTNs associated with LP, QTN A02_75551547 was presented in three planting environments (AY-2014, SHZ-2014, and SHZ-2015); two QTNs A02_74713290 and A12_7739289 were detected to be associated with LP in SHZ-2014 and SHZ-2015; QTN A07_16082894 was associated with LP in SHZ-2014 (Table 2). Importantly, three QTNs (A02_75551547, A02_74713290, and A12_7739289) were simultaneously identified in two or more environments, and had a high -lg*P* value (4.30–5.16) and a large explanation of the total phenotypic variance (10.78–15.54%).

**Table 2:** The significant QTNs associated with lint percentage (LP) *via* SL-GWAS methods.

QTNs	Chr.	Pos.	Env.	-Lg*P*	*R*^2^ (%)
A02_74713290	A02	74713290	SHZ-2014	4.52	11.59
			SHZ-2015	4.50	11.24
A02_75551547	A02	75551547	AY-2014	5.05	15.54
			SHZ-2014	4.52	14.07
			SHZ-2015	4.78	15.32
A07_16082894	A07	16082894	SHZ-2014	4.16	10.14
A12_7739289	A12	7739289	SHZ-2014	4.30	10.78
			SHZ-2015	5.16	13.58

*QTNs, quantitative trait nucleotides; Env., environment; Chr., chromosome; Pos., position; AY, Anyang; SHZ, Shihezi; the -lg*P*, the significance level; *R*^*2*^ (%), the percentage of phenotypic variation explained by each QTNs; SL-GWAS methods, single-locus GWAS.*

Using six ML-GWAS methods including mrMLM, FASTmrMLM, FASTmrEMMA, pLARmEB, ISIS EM-BLASSO, and pKWmEB, we totally identified 45 QTNs for LP after removing duplicates. Most QTNs (20) were detected by pKWmEB, followed by FASTmrMLM (18), pLARmEB (17), mrMLM (16), ISIS EM-BLASSO (13), and FASTmrEMMAQTNs (5) (Supplementary Table S3). To improve accuracy of the identified QTNs, the significant SNPs, which were simultaneously detected through three or more multi-locus methods, were considered as the steady and reliable QTNs. Finally, 11 steady and reliable QTNs for LP were screened out by three or more multi-locus methods (Table 3). Among these, six and five of them were respectively positioned on At- and Dt- chromosome, and three significant QTNs were simultaneously detected *via* five multi-locus methods. For example, the QTN A02_74713290 was probed using five multi-locus methods (mrMLM, FASTmrMLM, ISIS EM-BLASSO, pLARmEB, and pKWmEB) in SHZ-2015; the significant QTN D12_36345100 was detected in AY-2015 by using five ML-GWAS methods including mrMLM, FASTmrMLM, FASTmrEMMA, ISIS EM-BLASSO, and pKWmEB. Additionally, we observed three QTNs (A02_74713290, A02_75551547, and A05_12957926) were contemporaneously found in two planting environments. For instance, the QTN A05_12957926 was simultaneously associated with LP in two growing environments (AY-2014 and SHZ-2014) using three or more ML-GWAS methods, and explained 4.62–9.58% of total phenotypic variance. Most meaningfully, two QTNs were also found to be associated with LP in two of the four planting conditions. In detail, one QTN A02_75551547 was simultaneously presented in AY-2014 and SHZ-2014, with the highest LOD value (8.71) and the largest explanation of the total phenotypic variance (14.35%); and the other QTN A02_74713290 was simultaneously associated with LP in SHZ-2014 and SHZ-2015, and explained 5.52–11.52% of total phenotypic variance.

**Table 3:** The significant LP-QTNs detected simultaneously by using three or more ML-GWAS methods.

QTNs	Chr.	Env.	Pos.	LOD	*R*^2^ (%)	ML-GWAS methods
A01_95327880	A01	SHZ-2015	95.33	3.19–5.83	7.38–12.54	2, 4, 6
A02_74713290	A02	SHZ-2014	74.71	3.48–5.25	5.52–7.50	1, 2, 5
		SHZ-2015	74.71	4.73–6.05	9.09–11.52	1, 2, 4, 5, 6
A02_75551547	A02	AY-2014	75.55	6.95–8.71	8.41–14.28	1, 2, 5
		SHZ-2014	75.55	5.45–7.94	8.98–14.35	1, 2, 4
A05_12957926	A05	AY-2014	12.96	3.60–6.87	5.25–9.16	1, 2,3
		SHZ-2014	12.96	3.35–8.28	4.62–9.58	1, 2, 3, 4, 6
A05_40135551	A05	SHZ-2015	40.14	3.69–4.46	2.42–5.64	2, 5, 6,
A09_68324802	A09	SHZ-2015	68.32	3.02–3.79	3.96–5.93	3, 4, 6
D03_15827361	D03	AY-2015	15.83	3.19–5.84	6.45–9.76	1, 4, 6
D03_46455827	D03	SHZ-2014	46.46	3.12–4.66	1.81–4.27	3, 4, 5
D05_58804007	D05	AY-2015	58.8	3.65–5.14	7.23–10.65	2, 4, 5, 6
D12_36345100	D12	AY-2015	36.35	3.09–6.08	4.91–13.69	1, 2, 3, 4, 6
D13_55027992	D13	AY-2014	13.55	3.54–3.61	2.08–6.50	1, 2, 5

*QTNs, quantitative trait nucleotides; Env., environment; Chr., chromosome; Pos., position; AY, Anyang; SHZ, Shihezi. LOD, the significance levels; *R*^*2*^, the percentage of phenotypic variation explained by each QTN; ML-GWAS, multi-locus GWAS; mrMLM, FASTmrMLM, FASTmrEMMA, ISIS EM-BLASSO, pLARmEB and pKWmEB are marked by 1 to 6, respectively.*

In summary, we found that more QTNs were detected by applying multi-locus models than single-locus models. For instance, more than 13 QTNs were identified by whichever ML-GWAS methods with the exception of FASTmrEMMA, whereas only 4 QTNs were obtained *via* SL-GWAS method in the looser *P*-value threshold (5/total SNPs used). Interestingly, two QTNs (A02_74713290 and A02_75551547) were simultaneously identified *via* both SL- and ML-GWAS. Using the MLM of SL-GWAS, both A02_74713290 and A02_75551547 were detected in two or more cultivation environments. Similarly, the above two QTNs were also found to be associated with LP by three or more multi-locus methods in two planting conditions. These results demonstrated that the SNP loci A02_74713290 and A02_75551547 were steady and reliable main-effect QTNs for LP in Chinese early-maturity upland cotton.

### Allelic Variations for Four Significant QTNs

**FIGURE 2:**
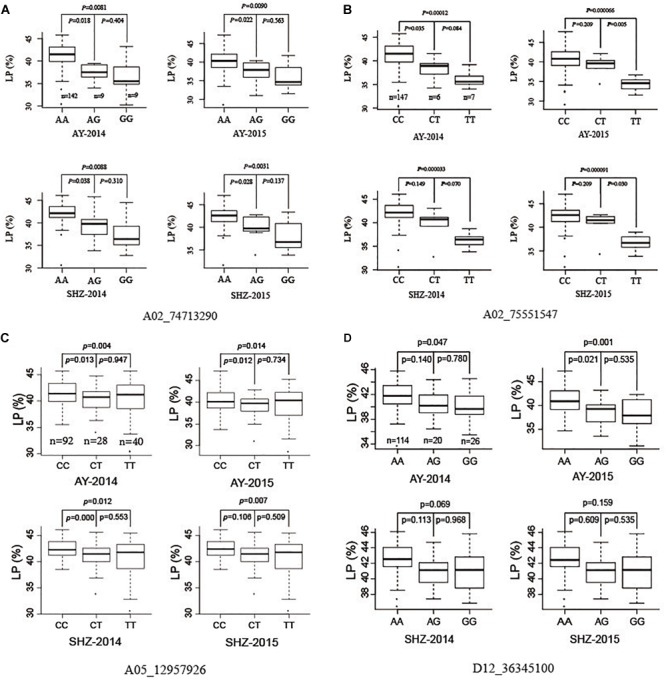
Box plots of lint percentage (LP) of three genetic variations for four peak QTNs A02_74713290 **(A)**, A02_75551547 **(B)**, A05_12957926 **(C)**, and D12_36345100 **(D)**.

To investigate further allelic variations for LP, we focused four QTNs (A02_74713290, A02_75551547, A05_12957926, and D12_36345100) that were associated significantly with LP in two planting conditions, or detected by using five ML-GWAS methods. The peak QTN A02_74713290 presented three allelic variations (AA, AG, and GG), and the LP values of 142 accessions with the allelic variation AA were significantly higher than those of nine accessions with the allele AG (*P* ≤ 0.05), and were significantly higher than those of nine accessions with the allele GG (*P* ≤ 0.01) in the four growing environments (Figure 2A). Analogously, the other striking QTN A02_75551547 had three types of allelic variation CC, CT, and TT, respectively, where the LPs of germplasms with the allele CC were significantly higher than those with the allele TT (*P* ≤ 0.01) in all the planting environments (Figure 2B). The QTN A05_12957926 had three allelic variations (CC, CT, and TT), and the average LP of 92 cotton accessions with CC type were 41.55, 40.25, 42.45, and 42.51% in AY-2014, AY-2015, SHZ-2014, and SHZ-2015, respectively, significantly higher than those of 40 accessions with TT type (*P* ≤ 0.01) (Figure 2C). For the QTN D12_36345100, although the mean LP values of114 accessions with AA type were higher than those of 26 accessions with GG type, they did not reach the significant level (*P* ≤ 0.05) on statistics in two growing environments of SHZ (Figure 2D). Considering the breeding objective of cotton production, four allelic variations A02_74713290-AA, A02_75551547-CC, A05_12957926-CC, and D12_36345100-AA should be favorable allelic variations, whereas A02_74713290-GG, A02_75551547-TT, A05_12957926-TT, and D12_36345100-GG were the unfavorable allelic variations.

**FIGURE 3:**
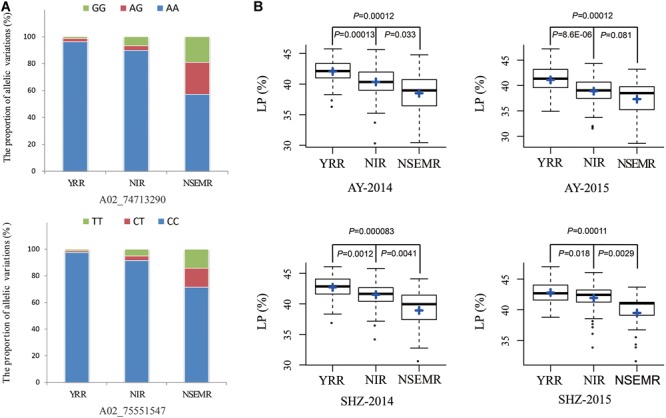
Identification of the favorable allelic variations for two peak QTNs. **(A)** The distribution frequencies of the favorable allelic variations in three geographic areas (YRR, NIR, and NSEMR). **(B)** Box plots of lint percentage (LP) of the different allelic variations for two peak QTNs in three geographic areas.

Additionally, to gain insight into the geographic distribution of favorable allelic variations, two peak QTNs (A02_74713290 and A02_75551547) of them were selected, and the proportions of three allelic variations were compared among YRR, NIR, and NSEMR. We found that the accessions from YRR accounted for a larger proportion of two favorable allelic variations than those from NIR and NSEMR, and the unfavorable allelic variations accounted for the largest ratio in the accessions from NSEMR (Figure 3A). Consistently, the accessions from YRR showed significantly higher LP than those from NIR and NSEMR in all the planting environments (*P* ≤ 0.01, Figure 3B). These data indicated that there was a close relationship between the proportion of favorable allelic variations and LP phenotype of cotton accessions from the different growing areas.

### Prediction of Candidate Genes for LP

**FIGURE 4:**
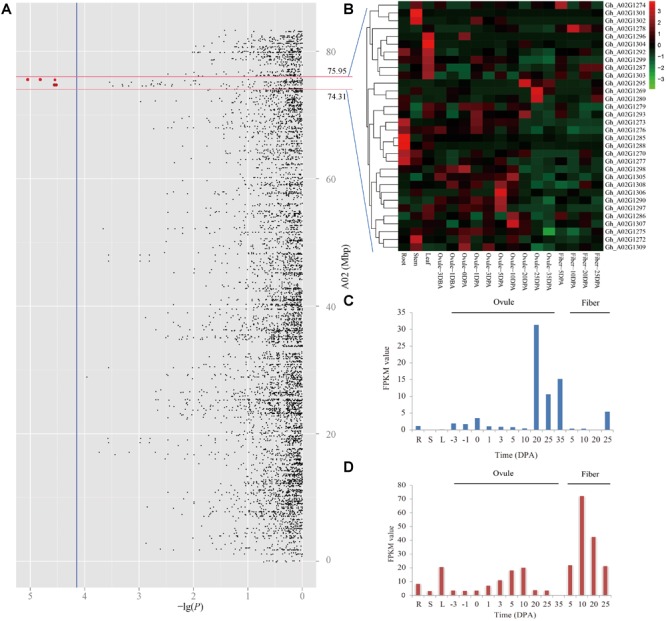
Prediction of candidate genes for of lint percentage (LP). **(A)** Manhattan plots for LP on chromosome A02, the red dots indicates two peak QTNs (A02_74713290 and A02_75551547) which were detected by using SL-GWAS methods in the different planting environments. **(B)** Heatmap of expression level of 32 genes among 17 upland cotton tissues, the red represents high expression, and the green shows low expression. Expression levels of *Gh_A02G1295* **(C)** and *Gh_A02G1278* **(D)** in different tissues, including the root (R), stem (S), and leaf (L), in ovule- and fiber-development stages based on the FPKM value.

Due to the panel LD decay rate of 400 kb and the mean marker density with one SNP per 28.10 kb, there were enough dense SNPs to detect the significantly associated QTNs (Su et al., 2018). In the present study, the genomic region within ± 400 kb of these two peak QTNs (A02_74713290 and A02_75551547) on chromosome A02 might be a steady major-effect QTL controlling LP in early-maturity upland cotton. Therefore, it was applied to the authentication of candidate genes. The physical distance between A02_74713290 and A02_75551547 was a small region of approximately 800 kb; thus, only one target region ranging from 74.31 to 75.95 Mbp on chromosome A02 was used for identifying candidate genes (Figure 4A). There were totally 42 genes in the genomic region A02: 74.31–75.95 Mbp (Supplementary Table S4). The RNA-seq data showed that 32 of them were expressed genes among 17 upland cotton tissues, according to the normalized FPKM values of the genes (Figure 4B). The GO enrichment analysis showed that these expressed genes mainly participated in glutathione metabolic process and gamma-glutamyl transferase activity (Supplementary Figure S1). Among the expressed genes, three genes (*Gh_A02G1269, Gh_A02G1280*, and *Gh_A02G1295*) had the highest expression in ovules at 20 and 25 DPA, which decreased LP by increasing seed weight during ovule development from 20 to 25 DPA. For instance, the expression of *Gh_A02G1295* in the late ovule-development stages was more than fivefold higher than that in fibers (Figure 4C). Moreover, *Gh_A02G1278* showed to be preferentially expressed in the fibers rather than other organs, which improved LP by increasing fiber output during fiber development from 10 to 20 DPA (Figure 4D). These results imply that the four genes (*Gh_A02G1269, Gh_A02G1278, Gh_A02G1280*, and *Gh_A02G1295*) might be related to LP of early-maturity upland cotton.

For the above four potential candidate genes for LP, their biological functions were annotated through bioinformatics analyses and GO items (Table 4). Three of them had explicit annotation about their biological functions in *Arabidopsis*. *Gh_A02G1269* is homologous to *Arabidopsis AT4G13830*, which encodes chaperone protein dnaJ 20, a key regulator of isopentenyl diphosphate biosynthetic process (Banerjee et al., 2013). *Gh_A02G1278* is the homologous to *Arabidopsis AT1G15100*, which encodes E3 ubiquitin-protein ligase RHA2A, involved in the positive regulation of abscisic acid (ABA) signaling and responses to salt and osmotic stresses during seed germination and early seedling development (Bu et al., 2009). *Gh_A02G1295* is homologous to *Arabidopsis AT5G09970* which is annotated as a cytochrome *P450* gene involved in multicellular organism development, oxidation-reduction process, regulation of growth, and regulation of meristem growth. In *Arabidopsis*, its ortholog *CYP78A5* plays a role in regulating relative growth of the shoot apical meristem and plant organs *via* a non-cell-autonomous signal (Wang et al., 2008). The possible biological functions of the genes provided some useful information for confirming their effects for cotton LP.

**Table 4:** The biological function annotations of four potential candidate genes for LP.

Gene ID	Gene name	Homologous gene	Description	GO annotations
*Gh_A02G1269*	*ATJ20*	*AT4G13830*	Chaperone protein dnaJ 20, chloroplastic	Regulation of isopentenyl diphosphate biosynthetic process, mevalonate-independent pathway
*Gh_A02G1278*	*RHA2A*	*AT1G15100*	E3 ubiquitin-protein ligase RHA2A	E3 ubiquitin-protein ligase involved in the positive regulation of abscisic acid (ABA) signaling and responses to salt and osmotic stresses during seed germination and early seedling development
*Gh_A02G1280*		NA	NA	NA
*Gh_A02G1295*	*CYP78A7*	*AT5G09970*	Cytochrome P450 78A7	Multicellular organism development, oxidation-reduction process, regulation of growth, and regulation of meristem growth

## Discussion

With the rapid development of high-throughput sequencing and molecular quantitative genetics, many GWAS methods have appeared for the genetic decryption of complex quantitative traits in plants (Feng et al., 2016). However, the SL-GWAS analysis approaches, which based on a fixed-SNP-effect MLM were mainly applied in the previous studies. Due to the stringent *P* threshold of SL-GWAS (*P* = 0.05/*n, n* is the total number of SNPs), some crucial QTNs might be lost in MLM, particularly small-effect QTNs (Wang et al., 2016). Although usage of high and stringent thresholds can reduce false positive rates, it results in missing some associated QTNs. In the recent 10 years, some multi-locus models, such as Bayesian LASSO (Yi and Xu, 2008), penalized Logistic regression (Hoggart et al., 2008), and EBAYES LASSO (Wen et al., 2015), had emerged for improving the efficiency of QTN detection. An obvious merit of these multi-locus models is that Bonferroni correction is not required and more QTNs can be detected than single-locus models. In particular, six recently developed ML-GWAS models including mrMLM (Wang et al., 2016), ISIS EM-BLASSO (Tamba et al., 2017), FASTmrEMMA (Wen et al., 2018), pLARmEB (Zhang et al., 2017), FASTmrMLM (Tamba and Zhang, 2018), and pKWmEB (Ren et al., 2018), have been proved to have more advantages for QTL detection than the single-locus methods. Some of these ML-GWAS models had been applied in rice (Cui et al., 2018), maize (Xu et al., 2018), wheat (Peng et al., 2018), cotton (Li et al., 2018; Su et al., 2018), soybean (Zhang et al., 2018), and barley (Hu et al., 2018). As the heredity of quantitative traits is complex and the number of SNPs is huge, it is better to simultaneously use multiple methods for GWAS. Therefore, to detect the stable and dependable QTNs, it is a requirement for combination of the SL- and ML-GWAS methods. Several examples can be found in recent studies. A total of 342 QTNs controlling fiber quality traits were detected *via* three SL-GWAS and three ML-GWAS models in upland cotton (Li et al., 2018). In addition, one single-locus method (GEMMA) and three multi-locus methods (FASTmrEMMA, FarmCPU, and LASSO) were used and 60 QTNs for starch pasting properties were identified by GWAS in maize (Xu et al., 2018). In the study, four significant QTNs were identified by single-locus model in the looser *P* threshold of SL-GWAS (*P* = 5/the total number of SNPs); meanwhile, applying six multi-locus models (mrMLM, FASTmrEMMA, pLARmEB, ISIS EM-BLASSO, pKWmEB, and FASTmrMLM), a total of 11 significant QTNs were simultaneously found to be associated with LP by at least three ML-GWAS methods. This study showed that more QTNs were detected using multi-locus models than using single-locus models, and these two major LP-QTNs with the highest -lg*P* value and a large explanation of the total phenotypic variance were simultaneously identified in both single- and multi-locus models. Our findings also demonstrated that employing integrated SL- and ML-GWAS models, led to improving power and accuracy levels for QTN detections.

In China, early-maturity upland cotton is more suitable for the high-profit production based on mechanical harvesting and double cropping. Thus, it becomes increasingly necessary for cotton producers (Su et al., 2018). But the trouble is that lint yield per plant of early-maturity varieties is strikingly lower than that of middle- and late-maturity ones. Although lint yield of early-maturity cotton can be improved by increasing the boll number per unit area at high-density planting, excellent varieties are beneficial to improving lint yield. In three main components of lint yield, LP has a high heritability and stability (Su et al., 2016a). Hence, it is extraordinarily vital for high-yield breeding programs to dissect the genetic basis controlling cotton LP by GWAS. In the previous studies, some GWAS of upland cotton accessions, based on the next-generation genome sequencing and SNP arrays, had been performed (Fang et al., 2017; Huang et al., 2017; Ma et al., 2018, 2019). The LP-QTNs, such as A02:79153947 and D08:3040023 (Fang et al., 2017), D08: 63485399 and D08: 63648326 (Huang et al., 2017), D02:131937, D02:132520, and D02:133540 (Ma et al., 2018), and D02:2254167 (Ma et al., 2019), were detected *via* GWAS (Table 5). In these investigations, the different QTNs have been identified to be associated with LP by using the varying association panels, and these SNP loci are primarily distributed on chromosomes A02, D02, and D08. In our study, two main-effect LP-QTNs A02_74713290 and A02_75551547 were identified in the panel consisting of 160 early-maturity upland cotton accessions *via* SL- and ML- GWAS, and these two QTNs also were positioned on chromosome A02. It was believed that A02_74713290 and A02_75551547 were reliable and stable QTNs for LP because they could be simultaneously presented in two or more planting environments and by multiple GWAS methods. Compared with those of previous GWAS results, we thought two significant QTNs (A02_74713290 and A02_75551547), which were detected in early-maturity upland cotton, may be two novel QTNs for LP.

**Table 5:** Results of the LP-QTNs identified *via* GWAS in previous studies.

QTNs	Chr.	Methods developing SNPs	Candidate genes	References
A02:79153947	A02	Genome-wide resequencing	*Gh_A02G1392*	Fang et al., 2017
D02:131937, D02:132520, D02:133540	D02	Genome-wide resequencing	*Gh_D02G0025*	Ma et al., 2018
D02:2254167	D02	Genome-wide resequencing	*Gh_D02G0203*	Ma et al., 2019
D08: 63485399,				
D08: 63648326	D08	CottonSNP63K array	*Gh_D08G2369, Gh_D08G2376*	Huang et al., 2017
D08:3040023	D08	Genome-wide resequencing	*Gh_D08G0312*	Fang et al., 2017

*QTNs, quantitative trait nucleotides; Chr., chromosome; SLAF-seq, specific-locus amplified fragment sequencing.*

Similarly, some potential candidate genes for LP had been forecasted in the adjacent region of the peak SNPs in this study. The four potential candidate genes were predicted in the adjacent region of the two major QTNs (A02_74713290 and A02_75551547) and they were specially and highly expressed in ovules or fibers. We suggested that these genes might be closely related to cotton LP by regulating the proportion of seed weight and fiber yield. Of these four candidate genes, *Gh_A02G1295* is annotated as cytochrome *P450* or *CYP78A7*. Its homologous genes caused a shortening of the plastochron in *Arabidopsis* (*ALTERED MERISTEM PROGRAM1, AMP1*) (Conway and Poethig, 1997), rice (*PLASTOCHRON1, PLA1*) (Itoh et al., 1998), and maize (*TERMINAL EAR1, TE1*) (Veit et al., 1998). Other studies demonstrated that *AMP1* orthologs *CYP78A5* and *CYP78A7* affect plastochron length and cell division rate and meristem size (Wang et al., 2008). Hence, we speculated that the candidate gene *Gh_A02G1295* might decrease LP of early-maturity upland cotton by increasing cell division rate and meristem size of ovule during seed development from 20 to 25 DPA. Even so, the biological function confirmation of these candidate genes is required in future studies.

## Conclusion

In the study, SL- and ML-GWAS methods were used to identify QTNs associated with LP in early-maturity upland cotton. We identified 4 and 45 QTNs associated significantly with LP *via* one single-locus method and six multi-locus methods. Two of these QTNs (A02_74713290 and A02_75551547) were simultaneously found *via* both one SL-GWAS and there or more ML-GWAS methods. Four potential candidate genes (*Gh_A02G1269, Gh_A02G1278, Gh_A02G1280*, and *Gh_A02G1295*) were predicted by RNA-seq in the flanking region of these two peak QTNs. The findings demonstrated that the detected QTNs and candidate genes might be closely related to LP of early-maturity cotton, and that a comprehensive application of SL- and ML-GWAS methods could help improve the detection power and accuracy. The QTNs and candidate genes for LP identified in this study have laid a foundation for cultivating novel cotton varieties with earliness and high lint yield in the future.

## Data Availability

The SLAF-seq datasets for the early-maturity upland cotton lines are available in the Sequence Read Archive (http://www. ncbi.nlm.nih.gov/bioproject/PRJNA314284/) (SRP071133 under the accession number: PRJNA314284).

## Author Contributions

JS and XN designed the research program. JS, CW, and JW analyzed the data and conducted the GWAS. JS, JL, and XN performed the field trial to identify the traits. JS and FH wrote the manuscript. All authors read and approved the manuscript.

## Conflict of Interest Statement

The authors declare that the research was conducted in the absence of any commercial or financial relationships that could be construed as a potential conflict of interest.

## Footnotes

1 http://www.ncbi.nlm.nih.gov/sra/?term=PRJNA248163

2 https://cottonfgd.org/

## Abbreviations

ANOVA: analysis of variance
AY: Anyang
BW: boll weight
CV: coefficients of variance
DPA: days post anthesis
FPKM: fragments per kilobase of transcript per million fragments mapped reads
GO: gene ontology
GWAS: genome-wide association study
LD: linkage disequilibrium
LP: lint percentage
LW: lint weight
MAF: minor allele frequency
ML-GWAS: multi-locus GWAS
MLM: mixed linear model
NSEMR: Northern Specific Early-Maturity Region
NIR: Northwest Inland Region
QTL: quantitative trait loci
QTNs: quantitative trait nucleotides
SHZ: Shihezi
SLAF-seq: specific-locus amplified fragment sequencing
SNP: single nucleotide polymorphism
SL-GWAS: single-locus GWAS
YRR: Yellow River Region
