# Supplementary material for: Genetic Detection of Lint Percentage Applying Single-Locus and Multi-Locus Genome-Wide Association Studies in Chinese Early-Maturity Upland Cotton
Source: Front Plant Sci. 2019 Aug 2;10:964. doi: 10.3389/fpls.2019.00964 (PMC6688134; doi:10.3389/fpls.2019.00964)
Supplement: Supplementary file 1 [file Table_1.docx]

| Table S1 Information of 160 early-maturity upland cotton accessions |
| --- |
| \| No. \| Varieties \| Growing areas \| No. \| Varieties \| Growing areas \| \| --- \| --- \| --- \| --- \| --- \| --- \| \| 1 \| zhongzao0191 \| YRR \| 41 \| Jinmian23 \| YRR \| \| 2 \| zhongzao0418 \| YRR \| 42 \| Jinmian5 \| YRR \| \| 3 \| zhongzao0618 \| YRR \| 43 \| liaomian10 \| NSEMR \| \| 4 \| zhongzao0712 \| YRR \| 44 \| liaomian17 \| NSEMR \| \| 5 \| zhongzao0811 \| YRR \| 45 \| liaomian5 \| NSEMR \| \| 6 \| zhongzao0822 \| YRR \| 46 \| liaomian6 \| NSEMR \| \| 7 \| zhongzao1832 \| YRR \| 47 \| liaomian7 \| NSEMR \| \| 8 \| zhongzao1239 \| YRR \| 48 \| liaomian9 \| NSEMR \| \| 9 \| PB12-1-10 \| YRR \| 49 \| liaomianduanjie \| NSEMR \| \| 10 \| PB12-1-7 \| YRR \| 50 \| lu154 \| YRR \| \| 11 \| PB12-1-8 \| YRR \| 51 \| lu890 \| YRR \| \| 12 \| zhongzao1476 \| YRR \| 52 \| lumian2153 \| YRR \| \| 13 \| zhongzao1222 \| YRR \| 53 \| nongken5 \| NIR \| \| 14 \| zhongzao2201 \| YRR \| 54 \| shan70 \| YRR \| \| 15 \| SQ152201 \| YRR \| 55 \| shizao1 \| YRR \| \| 16 \| zhongzao2214 \| YRR \| 56 \| shizao2 \| YRR \| \| 17 \| SQ152224 \| YRR \| 57 \| shizao3 \| YRR \| \| 18 \| 2011SS \| YRR \| 58 \| xia13-7 \| YRR \| \| 19 \| QS2012-3 \| YRR \| 59 \| xia25 \| YRR \| \| 20 \| QS2012-4 \| YRR \| 60 \| xiazao1 \| YRR \| \| 21 \| 29-41 \| YRR \| 61 \| xiazao2 \| YRR \| \| 22 \| 29-42 \| YRR \| 62 \| xiazao3 \| YRR \| \| 23 \| 6426 \| YRR \| 63 \| xinluzao11 \| NIR \| \| 24 \| K640 \| YRR \| 64 \| xinluza \| NIR \| \| 25 \| N82 \| YRR \| 65 \| xinluzao3 \| NIR \| \| 26 \| baimian17 \| YRR \| 66 \| xinluzao42 \| NIR \| \| 27 \| chaoyangmian1 \| NSEMR \| 67 \| xinluzao45 \| NIR \| \| 28 \| Deltapine20 \| NSEMR \| 68 \| xinluzao4 \| NIR \| \| 29 \| Delfos97-047 \| NSEMR \| 69 \| xinluzao6 \| NIR \| \| 30 \| guannong1 \| NSEMR \| 70 \| xinluzao8 \| NIR \| \| 31 \| han2490 \| YRR \| 71 \| xinluzao9 \| NIR \| \| 32 \| han656 \| YRR \| 72 \| xinxiang368 \| YRR \| \| 33 \| han559 \| YRR \| 73 \| yu1335 \| YRR \| \| 34 \| han667 \| YRR \| 74 \| yumian12 \| YRR \| \| 35 \| han686 \| YRR \| 75 \| yuzao8E13 \| YRR \| \| 36 \| han9609 \| YRR \| 76 \| yuzao910 \| YRR \| \| 37 \| heishanmian1 \| NSEMR \| 77 \| yunzaoN177 \| YRR \| \| 38 \| Jingmian3 \| NSEMR \| 78 \| yunzaoN95 \| YRR \| \| 39 \| Jinmian10 \| YRR \| 79 \| zhong416 \| YRR \| \| 40 \| Jinmian21 \| YRR \| 80 \| zhong425-5 \| YRR \| |

| Table S1 Information of the 160 early-maturity upland cotton accessions (continued) |
| --- |
| \| No. \| Varieties \| Growing areas \| No. \| Varieties \| Growing areas \| \| --- \| --- \| --- \| --- \| --- \| --- \| \| 81 \| zhong716 \| YRR \| 121 \| xinluzao27 \| NIR \| \| 82 \| zhong751213 \| YRR \| 122 \| xinluzao28 \| NIR \| \| 83 \| CRI10 \| YRR \| 123 \| xinluzao29 \| NIR \| \| 84 \| CRI14 \| YRR \| 124 \| xinluzao30 \| NIR \| \| 85 \| CRI16 \| YRR \| 125 \| xinluzao32 \| NIR \| \| 86 \| CRI20 \| YRR \| 126 \| xinluzao33 \| NIR \| \| 87 \| CRI24 \| YRR \| 127 \| xinluzao34 \| NIR \| \| 88 \| CRI27 \| YRR \| 128 \| xinluzao35 \| NIR \| \| 89 \| CRI30 \| YRR \| 129 \| xinluzao37 \| NIR \| \| 90 \| CRI36 \| YRR \| 130 \| xinluzao38 \| NIR \| \| 91 \| han256 \| YRR \| 131 \| xinluzao39 \| NIR \| \| 92 \| CRI37 \| YRR \| 132 \| xinluzao40 \| NIR \| \| 93 \| CRI42 \| YRR \| 133 \| xinluzao41 \| NIR \| \| 94 \| CRI50 \| YRR \| 134 \| xinluzao46 \| NIR \| \| 95 \| CRI58 \| YRR \| 135 \| xinluzao47 \| NIR \| \| 96 \| CRI64 \| YRR \| 136 \| xinluzao48 \| NIR \| \| 97 \| CRI74 \| YRR \| 137 \| xinluzao49 \| NIR \| \| 98 \| liaomian23 \| NSEMR \| 138 \| xinluzao50 \| NIR \| \| 99 \| liaomian27 \| NSEMR \| 139 \| xinluzao51 \| NIR \| \| 100 \| liaomian28 \| NSEMR \| 140 \| xinluzao60 \| NIR \| \| 101 \| xinluzao2 \| NIR \| 141 \| huiyuan717 \| NIR \| \| 102 \| xinluzao10 \| NIR \| 142 \| yunzao219 \| YRR \| \| 103 \| xinluzao12 \| NIR \| 143 \| yunzao33-356 \| YRR \| \| 104 \| xinluzao13 \| NIR \| 144 \| jinmian2 \| NSEMR \| \| 105 \| xinluzao15 \| NIR \| 145 \| chaoyangmian2 \| NSEMR \| \| 106 \| xinluzao16 \| NIR \| 146 \| dunhuang77-116 \| NIR \| \| 107 \| xinluzao17 \| NIR \| 147 \| ganmian4 \| NIR \| \| 108 \| xinluzao18 \| NIR \| 148 \| guannongzaoC-50 \| NSEMR \| \| 109 \| xinluzao19 \| NIR \| 149 \| guannongchangzao14 \| NSEMR \| \| 110 \| xinluzao20 \| NIR \| 150 \| yanzao1 \| YRR \| \| 111 \| xinluzao21 \| NIR \| 151 \| yanzao2 \| YRR \| \| 112 \| xinluzao22 \| NIR \| 152 \| yiaojinmian6 \| NSEMR \| \| 113 \| xinluzao23 \| NIR \| 153 \| jinken69-2 \| NIR \| \| 114 \| xinluzao24 \| NIR \| 154 \| jinken148-39 \| NIR \| \| 115 \| yumian5 \| YRR \| 155 \| 611bo \| NIR \| \| 116 \| yumian18 \| YRR \| 156 \| dunmian1 \| NIR \| \| 117 \| bo425 \| NIR \| 157 \| dunmian2 \| NIR \| \| 118 \| kenN27-3 \| NIR \| 158 \| ganmian2 \| NIR \| \| 119 \| xinluzao25 \| NIR \| 159 \| keke1543 \| NIR \| \| 120 \| xinluzao26 \| NIR \| 160 \| xinluzao53 \| NIR \| |
| YRR, Yellow River Region; NIR, Northwest Inland Region; NSEMR, Northern Specific Early-Maturity Region. |
