# Supplementary material for: Genetic Detection of Lint Percentage Applying Single-Locus and Multi-Locus Genome-Wide Association Studies in Chinese Early-Maturity Upland Cotton
Source: Front Plant Sci. 2019 Aug 2;10:964. doi: 10.3389/fpls.2019.00964 (PMC6688134; doi:10.3389/fpls.2019.00964)
Supplement: Supplementary file 2 [file Table_2.docx]

Table S2 Analysis of variance (ANOVA) of the lint percentage (LP) of 160 lines

in four environments.

| Influencing factors | SS | df | MS | F | P-value | F crit |
| --- | --- | --- | --- | --- | --- | --- |
| Genotypes (G) | 12553.04 | 159 | 78.94994 | 32.22998 | 0 | 1.20614 |
| Environment (E) | 1480.346 | 3 | 493.4486 | 201.4421 | 5.3E-107 | 2.611855 |
| G×E interactions | 2156.499 | 477 | 4.520962 | 1.845606 | 1.83E-17 | 1.130681 |
