# Supplementary material for: Genetic Detection of Lint Percentage Applying Single-Locus and Multi-Locus Genome-Wide Association Studies in Chinese Early-Maturity Upland Cotton
Source: Front Plant Sci. 2019 Aug 2;10:964. doi: 10.3389/fpls.2019.00964 (PMC6688134; doi:10.3389/fpls.2019.00964)
Supplement: Supplementary file 3 [file Table_3.docx]

| Table S3 The significant QTNs for LP detected simultaneously by using each of ML-GWAS methods |
| --- |
| \| Methods \| QTNs \| Chromosome \| Pos. (bp) \| LOD \| R2 (%) \| Environments \| \| --- \| --- \| --- \| --- \| --- \| --- \| --- \| \| FASTmrEMMA \| A05_12957926 \| A05 \| 12957926 \| 4.67 \| 5.25 \| AY-2014 \| \|  \|  \|  \| 6.94 \| 9.15 \| SHZ-2014 \| \| A09_68324802 \| A09 \| 68324802 \| 3.79 \| 5.63 \| SHZ-2015 \| \| D03_46455827 \| D03 \| 46455827 \| 3.12 \| 3.04 \| SHZ-2014 \| \| D04_6999375 \| D04 \| 6999375 \| 4.25 \| 5.89 \| SHZ-2015 \| \| D12_36345100 \| D12 \| 36345100 \| 4.44 \| 7.34 \| AY-2015 \| \| FASTmrMLM \| A01_40199362 \| A01 \| 40199362 \| 3.98 \| 3.63 \| AY-2014 \| \| A01_95327880 \| A01 \| 95327880 \| 5.83 \| 12.54 \| SHZ-2015 \| \| A02_31026644 \| A02 \| 31026644 \| 3.61 \| 2.51 \| AY-2015 \| \| A02_74713290 \| A02 \| 74713290 \| 5.25 \| 7.50 \| SHZ-2014 \| \|  \|  \|  \| 5.12 \| 8.56 \| SHZ-2015 \| \| A02_75551547 \| A02 \| 75551547 \| 7.94 \| 14.35 \| SHZ-2014 \| \| A05_12957926 \| A05 \| 12957926 \| 6.87 \| 9.16 \| AY-2014 \| \|  \|  \|  \| 6.14 \| 8.08 \| SHZ-2014 \| \| A05_40135551 \| A05 \| 40135551 \| 3.69 \| 2.42 \| SHZ-2015 \| \| A06_98660253 \| A06 \| 98660253 \| 3.64 \| 3.84 \| SHZ-2015 \| \| A07_58768843 \| A07 \| 58768843 \| 4.11 \| 4.75 \| SHZ-2014 \| \| A12_82893388 \| A12 \| 82893388 \| 3.66 \| 3.84 \| SHZ-2015 \| \| A13_67617736 \| A13 \| 67617736 \| 3.41 \| 3.61 \| SHZ-2015 \| \| A13_77331324 \| A13 \| 77331324 \| 3.97 \| 3.98 \| SHZ-2015 \| \| D05_58804007 \| D05 \| 58804007 \| 5.14 \| 19.65 \| AY-2015 \| \| D06_60052939 \| D06 \| 60052939 \| 3.66 \| 3.35 \| AY-2014 \| \| D07_46992517 \| D07 \| 46992517 \| 6.42 \| 7.37 \| SHZ-2014 \| \| D07_46992517 \| D07 \| 46992517 \| 6.54 \| 7.68 \| AY-2014 \| \| D12_36345100 \| D12 \| 36345100 \| 5.31 \| 7.67 \| AY-2015 \| \| D13_55027992 \| D13 \| 55027992 \| 3.61 \| 4.16 \| AY-2014 \| \| ISIS EM-BLASSO \| A01_95327880 \| A01 \| 95327880 \| 3.19 \| 7.38 \| SHZ-2015 \| \| A02_29042832 \| A02 \| 29042832 \| 3.35 \| 10.80 \| SHZ-2015 \| \| A02_74713290 \| A02 \| 74713290 \| 4.73 \| 10.20 \| SHZ-2015 \| \| A02_75551547 \| A02 \| 75551547 \| 5.70 \| 14.28 \| AY-2014 \| \|  \|  \|  \| 5.46 \| 10.17 \| SHZ-2014 \| \| A05_12957926 \| A05 \| 12957926 \| 3.35 \| 4.62 \| SHZ-2014 \| \| A09_68324802 \| A09 \| 68324802 \| 3.02 \| 3.96 \| SHZ-2015 \| \| D03_15827361 \| D03 \| 15827361 \| 3.86 \| 11.45 \| AY-2015 \| \| D03_46455827 \| D03 \| 46455827 \| 3.91 \| 4.27 \| SHZ-2014 \| \|  \|  \|  \| 5.50 \| 7.27 \| SHZ-2015 \| \| D04_4649030 \| D04 \| 4649030 \| 3.43 \| 5.58 \| AY-2015 \| \| D05_58804007 \| D05 \| 58804007 \| 4.06 \| 12.67 \| AY-2015 \| \| D10_61333720 \| D10 \| 61333720 \| 4.40 \| 11.84 \| AY-2014 \| \| D12_2828783 \| D12 \| 2828783 \| 4.13 \| 4.88 \| AY-2015 \| \| D12_36345100 \| D12 \| 36345100 \| 3.09 \| 4.91 \| AY-2015 \| \| mrMLM \| A01_40199362 \| A01 \| 40199362 \| 3.05 \| 5.77 \| AY-2014 \| \| A02_48902032 \| A02 \| 48902032 \| 4.75 \| 20.69 \| SHZ-2015 \| \| A02_74713290 \| A02 \| 74713290 \| 4.90 \| 9.09 \| SHZ-2015 \| \|  \|  \|  \| 4.45 \| 7.22 \| SHZ-2014 \| \| A02_75551547 \| A02 \| 75551547 \| 6.95 \| 14.27 \| AY-2014 \| \|  \|  \|  \| 5.67 \| 8.98 \| SHZ-2014 \| \| A05_12957926 \| A05 \| 12957926 \| 3.61 \| 6.93 \| AY-2014 \| \|  \|  \|  \| 7.85 \| 19.58 \| SHZ-2014 \| \| A06_1228293 \| A06 \| 1228293 \| 3.20 \| 3.68 \| AY-2014 \| \| A08_95893154 \| A08 \| 95893154 \| 4.29 \| 8.04 \| SHZ-2015 \| \| A12_82893388 \| A12 \| 82893388 \| 3.02 \| 9.25 \| SHZ-2015 \| \| A13_77331324 \| A13 \| 77331324 \| 4.09 \| 5.39 \| SHZ-2015 \| \| D03_15827361 \| D03 \| 15827361 \| 5.84 \| 19.76 \| AY-2015 \| \| D04_6999375 \| D04 \| 6999375 \| 3.64 \| 6.21 \| SHZ-2015 \| \| D07_46992517 \| D07 \| 46992517 \| 6.85 \| 10.68 \| AY-2014 \| \|  \|  \|  \| 4.43 \| 6.35 \| SHZ-2014 \| \| D07_51699607 \| D07 \| 51699607 \| 3.49 \| 8.11 \| SHZ-2015 \| \| D11_36722656 \| D11 \| 36722656 \| 7.20 \| 31.80 \| AY-2015 \| \| D12_36345100 \| D12 \| 36345100 \| 4.23 \| 9.64 \| AY-2015 \| \| D13_55027992 \| D13 \| 55027992 \| 3.58 \| 6.50 \| AY-2014 \| \| pKWmEB \| A01_95327880 \| A01 \| 95327880 \| 3.41 \| 11.99 \| SHZ-2015 \| \| A02_74713290 \| A02 \| 74713290 \| 5.64 \| 11.52 \| SHZ-2015 \| \| A05_12957926 \| A05 \| 12957926 \| 8.28 \| 13.24 \| SHZ-2014 \| \| A05_40135551 \| A05 \| 40135551 \| 4.46 \| 5.64 \| SHZ-2015 \| \| A05_87303928 \| A05 \| 87303928 \| 3.50 \| 7.38 \| SHZ-2014 \| \| A06_100588002 \| A06 \| 100588002 \| 4.85 \| 17.62 \| AY-2014 \| \| A06_1228293 \| A06 \| 1228293 \| 3.09 \| 7.41 \| AY-2014 \| \| A06_31972362 \| A06 \| 31972362 \| 3.57 \| 14.57 \| AY-2014 \| \| A07_49513094 \| A07 \| 49513094 \| 4.68 \| 12.87 \| SHZ-2014 \| \| A09_68324802 \| A09 \| 68324802 \| 3.27 \| 5.93 \| SHZ-2015 \| \| A12_81352077 \| A12 \| 81352077 \| 4.84 \| 20.12 \| SHZ-2014 \| \|  \|  \|  \| 5.86 \| 21.19 \| SHZ-2015 \| \| A13_53171994 \| A13 \| 53171994 \| 3.09 \| 2.73 \| SHZ-2015 \| \| D01_44423928 \| D01 \| 44423928 \| 3.01 \| 4.36 \| SHZ-2015 \| \| D02_60098855 \| D02 \| 60098855 \| 5.48 \| 9.52 \| SHZ-2014 \| \| D03_15827361 \| D03 \| 15827361 \| 3.19 \| 16.57 \| AY-2015 \| \| D05_31870463 \| D05 \| 31870463 \| 3.48 \| 5.14 \| AY-2015 \| \| D05_58804007 \| D05 \| 58804007 \| 3.65 \| 17.24 \| AY-2015 \| \| D07_51699607 \| D07 \| 51699607 \| 3.68 \| 5.80 \| SHZ-2015 \| \| D12_2828783 \| D12 \| 2828783 \| 3.71 \| 7.75 \| AY-2015 \| \| D12_36345100 \| D12 \| 36345100 \| 6.08 \| 13.69 \| AY-2015 \| \| pLARmEB \| A02_29042832 \| A02 \| 29042832 \| 5.61 \| 14.80 \| SHZ-2015 \| \| A02_74713290 \| A02 \| 74713290 \| 3.48 \| 5.65 \| SHZ-2014 \| \|  \|  \|  \|  \| 10.33 \| SHZ-2015 \| \| A02_75551547 \| A02 \| 75551547 \| 8.71 \| 5.41 \| AY-2014 \| \| A05_40135551 \| A05 \| 40135551 \| 4.38 \| 4.19 \| SHZ-2015 \| \| A07_58768843 \| A07 \| 58768843 \| 3.17 \| 1.19 \| SHZ-2014 \| \| A09_4855359 \| A09 \| 4855359 \| 3.17 \| 0.86 \| AY-2014 \| \| A10_7601765 \| A10 \| 7601765 \| 5.28 \| 3.98 \| AY-2015 \| \| A12_75497023 \| A12 \| 75497023 \| 4.77 \| 3.37 \| SHZ-2014 \| \| D01_44032424 \| D01 \| 44032424 \| 4.48 \| 5.02 \| SHZ-2015 \| \| D02_60098855 \| D02 \| 60098855 \| 4.11 \| 1.38 \| SHZ-2014 \| \| D03_46455827 \| D03 \| 46455827 \| 4.28 \| 1.81 \| SHZ-2014 \| \|  \|  \|  \| 4.66 \| 4.77 \| SHZ-2015 \| \| D04_4649030 \| D04 \| 4649030 \| 3.42 \| 2.99 \| AY-2015 \| \| D04_6999375 \| D04 \| 6999375 \| 3.32 \| 4.42 \| SHZ-2015 \| \| D05_58804007 \| D05 \| 58804007 \| 4.73 \| 7.23 \| AY-2015 \| \| D09_10840263 \| D09 \| 10840263 \| 5.80 \| 4.61 \| AY-2015 \| \| D09_22615229 \| D09 \| 22615229 \| 4.59 \| 1.61 \| AY-2014 \| \| D13_55027992 \| D13 \| 55027992 \| 5.54 \| 2.08 \| AY-2014 \| |
