# Supplementary material for: Genetic Detection of Lint Percentage Applying Single-Locus and Multi-Locus Genome-Wide Association Studies in Chinese Early-Maturity Upland Cotton
Source: Front Plant Sci. 2019 Aug 2;10:964. doi: 10.3389/fpls.2019.00964 (PMC6688134; doi:10.3389/fpls.2019.00964)
Supplement: Supplementary file 4 [file Table_4.docx]

| Table S4 Information of 42 genes in the genomic region A02: 74.31-75.95 Mbp |
| --- |
| \| Gene ID \| Start \| End \| Gene Name \| Description \| \| --- \| --- \| --- \| --- \| --- \| \| *Gh_A02G1269* \| 74696904 \| 74697401 \| *ATJ20* \| Chaperone protein dnaJ 20, chloroplastic \| \| *Gh_A02G1270* \| 74764666 \| 74765731 \| *NEN4* \| Protein NEN4 \| \| *Gh_A02G1271* \| 74767650 \| 74768708 \| NA \| NA \| \| *Gh_A02G1272* \| 74781472 \| 74783399 \| NA \| L-ascorbate oxidase \| \| *Gh_A02G1273* \| 74858261 \| 74858819 \| *HIPP26* \| Heavy metal-associated isoprenylated plant protein 26 \| \| *Gh_A02G1274* \| 74904851 \| 74909046 \| *Tsr3* \| Ribosome biogenesis protein TSR3 homolog \| \| *Gh_A02G1275* \| 74912407 \| 74915325 \| *Acin1* \| Apoptotic chromatin condensation inducer in the nucleus \| \| *Gh_A02G1276* \| 74918186 \| 74921249 \| *ACIN1* \| Apoptotic chromatin condensation inducer in the nucleus \| \| *Gh_A02G1277* \| 74923422 \| 74923658 \| NA \| NA \| \| *Gh_A02G1278* \| 74962676 \| 74963170 \| *RHA2A* \| E3 ubiquitin-protein ligase RHA2A \| \| *Gh_A02G1279* \| 75026848 \| 75029266 \| *PRMT1* \| Probable protein arginine N-methyltransferase 1 \| \| *Gh_A02G1280* \| 75047394 \| 75047636 \| NA \| NA \| \| *Gh_A02G1281* \| 75099880 \| 75102118 \| *GGT1* \| Gamma-glutamyltranspeptidase 1 \| \| *Gh_A02G1282* \| 75105305 \| 75108105 \| *GGT1* \| Gamma-glutamyltranspeptidase 1 \| \| *Gh_A02G1283* \| 75108130 \| 75108429 \| *GGT1* \| Gamma-glutamyltranspeptidase 1 \| \| *Gh_A02G1284* \| 75114657 \| 75115505 \| *GGT1* \| Gamma-glutamyltranspeptidase 1 \| \| *Gh_A02G1285* \| 75156008 \| 75170790 \| *sll1770* \| Uncharacterized protein sll1770 \| \| *Gh_A02G1286* \| 75192373 \| 75198072 \| *At5g10020* \| Probable inactive receptor kinase At5g10020 \| \| *Gh_A02G1287* \| 75198717 \| 75202128 \| *CPR5* \| Protein CPR-5 \| \| *Gh_A02G1288* \| 75214741 \| 75215454 \| *NFYB3* \| Nuclear transcription factor Y subunit B-3 \| \| *Gh_A02G1289* \| 75272640 \| 75273521 \| NA \| NA \| \| *Gh_A02G1290* \| 75288857 \| 75291004 \| NA \| NA \| \| *Gh_A02G1291* \| 75321743 \| 75326882 \| NA \| NA \| \| *Gh_A02G1292* \| 75360814 \| 75361502 \| *43345* \| Stress enhanced protein 2, chloroplastic \| \| *Gh_A02G1293* \| 75380103 \| 75385905 \| *DPE1* \| 4-alpha-glucanotransferase DPE1, chloroplastic/amyloplastic \| \| *Gh_A02G1294* \| 75386743 \| 75390231 \| NA \| NA \| \| *Gh_A02G1295* \| 75497963 \| 75499674 \| *CYP78A7* \| Cytochrome P450 78A7 \| \| *Gh_A02G1296* \| 75535745 \| 75536094 \| *AHP5* \| Histidine-containing phosphotransfer protein 5 \| \| *Gh_A02G1297* \| 75553781 \| 75554242 \| NA \| NA \| \| *Gh_A02G1298* \| 75587871 \| 75589362 \| NA \| NA \| \| *Gh_A02G1299* \| 75613512 \| 75617600 \| *ABCF5* \| ABC transporter F family member 5 \| \| *Gh_A02G1300* \| 75618901 \| 75619463 \| NA \| NA \| \| *Gh_A02G1301* \| 75621065 \| 75624523 \| *WRKY51* \| Probable WRKY transcription factor 51 \| \| *Gh_A02G1302* \| 75725680 \| 75725946 \| *UGT75L6* \| Crocetin glucosyltransferase, chloroplastic \| \| *Gh_A02G1303* \| 75728522 \| 75730494 \| *VDE1* \| Violaxanthin de-epoxidase, chloroplastic \| \| *Gh_A02G1304* \| 75755053 \| 75757400 \| *WRKY13* \| Probable WRKY transcription factor 13 \| \| *Gh_A02G1305* \| 75794718 \| 75798278 \| *CURL3* \| Brassinosteroid LRR receptor kinase \| \| *Gh_A02G1306* \| 75805278 \| 75815841 \| *RABD2A* \| Ras-related protein RABD2a \| \| *Gh_A02G1307* \| 75872521 \| 75876568 \| *UDP-GALT2* \| UDP-galactose transporter 2 \| \| *Gh_A02G1308* \| 75915926 \| 75919071 \| NA \| NA \| \| *Gh_A02G1309* \| 75920433 \| 75922862 \| NA \| NA \| \| *Gh_A02G1310* \| 75924397 \| 75926234 \| NA \| NA \| |
