# Supplementary figures and images for: Genetic Detection of Lint Percentage Applying Single-Locus and Multi-Locus Genome-Wide Association Studies in Chinese Early-Maturity Upland Cotton
Source: Front Plant Sci. 2019 Aug 2;10:964. doi: 10.3389/fpls.2019.00964 (PMC6688134; doi:10.3389/fpls.2019.00964)

| 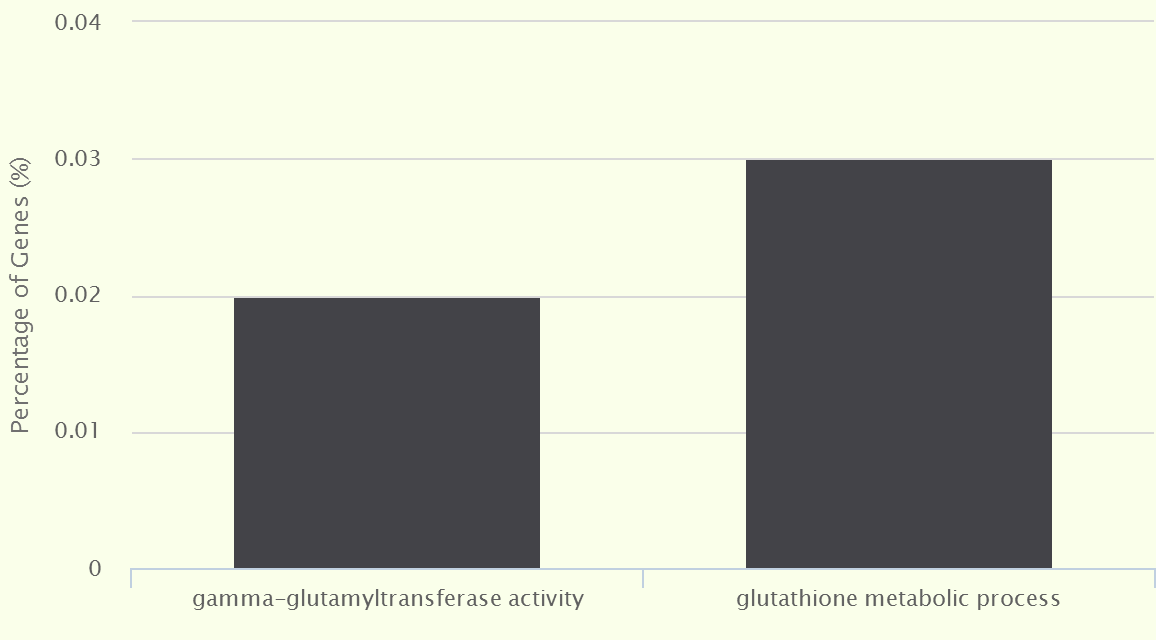 |
| --- |
| Figure S1 The GO enrichment analysis of 42 genes |

Supplement: Supplementary file 5 [file Data_Sheet_1.docx]
